# Slowing down in PNP: relationship of joint velocities in an arm-movement test and the fast TUG test

**DOI:** 10.1038/s41598-025-14463-5

**Published:** 2025-08-21

**Authors:** Isabelle D. Walz, Sarah Waibel, Vittorio Lippi, Albert Gollhofer, Christoph Maurer

**Affiliations:** 1 Department of Neurology and Neuroscience, Faculty of Medicine, Medical Center, University of Freiburg, Freiburg, Germany; 2 Department of Sport and Sport Science, University of Freiburg, Freiburg, Germany; 3 Institute of Digitalization in Medicine, Faculty of Medicine, Medical Center, University of Freiburg, Freiburg, Germany

**Keywords:** Motor control, Gait, Polyneuropathy, Instrumented timed up and go, TUG, Joint velocity, Whole-body motion capture, Peripheral nervous system, Neurological disorders

## Abstract

Polyneuropathy (PNP) is a prevalent neurological disorder that affects both upper and lower limbs leading to a decline in motor and sensory nerve function and consequently, to movement impairments. In our earlier work, we were able to demonstrate that PNP patients’ slower gait speed as compared to healthy subjects goes along with a relative speed reduction in all major joints of the body during gait, including the arms. It is not known yet, whether this speed reduction is confined to gait or whether it is a general phenomenon of cyclic movements in PNP, which may also show up in isolated goal directed arm movements. We aim here to assess joint speed traces across the body in PNP patients during gait and evaluate the relationship to joint velocities in a different task, i.e. goal directed repetitive arm movements. We compared performances of 20 PNP patients and 20 matched healthy individuals (CG) during (i) the walking sequence of the fast executed TUG test, and (ii) a fast repetitive goal-directed arm-movement test. We were able to reproduce the reduction in joint velocities across all relevant joints during walking. Moreover this reduction of joint velocities was almost evenly distributed across the velocity traces as a function of time. In the goal-directed arm-movement test, PNP patients showed again significantly lower joint velocities across all joints involved (upper body), compared to the CG, with mean and maximum velocities both significantly reduced. The mean velocities of arm-movement test and the degree of slowing observed in the arm-movement task was strongly correlated with TUG performance. Specifically, slower arm movements were associated with longer TUG times (ρ = -0.76, *p* < 0.001) and reduced gait speed (ρ = 0.79, *p* < 0.001). Arm movement slowing significantly correlated with clinical scales, including the Falls Efficacy Scale-International (FES-I, ρ = -0.55, *p* = 0.012) and the Performance-Oriented Mobility Assessment (POMA, ρ = 0.69, *p* < 0.001). We conclude that the evenly distributed reduction of joint speed as a function of time in PNP is not confined to gait, but also seems to appear in other repetitive motor tasks, here goal-directed arm movements. We assume that joint speed reduction is a general impairment in movement planning and execution of PNP patients. We speculate that this slowing is a consequence of less reliable/ less accurate sensory feedback in the sensorimotor control loop for movement execution. As a clinical application, this finding might help to quantify the amount of the impairment of the sensorimotor control loop in PNP when gait testing is not feasible. Moreover, it might lead to better targeted training strategies to enhance motor performance in PNP.

**Trial registration: **German Clinical Trials Register (DRKS), Identifier: DRKS00016999.

## Introduction

PNP are the most common disorders of the peripheral nervous system in adults with a current estimated prevalence of 5–8% that increases up to 13% in old age^1–5^. The incidence and prevalence are expected to increase in the coming years, placing a greater burden on healthcare resources^5–8^. PNP is characterized by damage to multiple peripheral nerves in a symmetrical, distal, length-dependent “glove and stocking” distribution^9^. The symptoms of polyneuropathies can be divided into sensory, motor, and autonomic. Sensory symptoms are the most common (> 85%), such as sensory disturbance with reduced sensation of pressure or touch (numbness, feeling of fur), increased sensitivity to pain (burning, stinging), hot-cold discomfort, and sensory ataxia (unsteady gait, falls)^4,10,11^.

Walking, a fundamental human activity that depends on coordination of the whole body, involves multiple joints and requires complex control mechanisms, including the precise placement of the feet in terms of time and position to generate propulsion with low energy consumption while maintaining a stable posture^1,12–14^. For individuals with PNP, this movement becomes a daunting task, mainly due to compromised proprioception^9,15–17^. PNP-induced somatosensory dysfunctions may lead to postural instability, balance, and gait problems^18,19^ with consequently higher fall rates^9,20–23,^limitations in daily mobility, and a reduced quality of life^24,25^. It is assumed that the reduced sensory feedback affects step-to-step trajectories of extremities^26,27^. Movements cannot be optimized in time, which in turn means that fewer adjustments take place and thus balance may be impaired during locomotion^26,27^. Patients who lack proprioceptive feedback also have deficits in motor coordination - particularly in the positioning of the extremities, force control, postural stability and the execution of coordinated movement sequences such as walking, but also in fine motor tasks of the upper extremity (e.g. closing buttons)^28,29^. Consequently, lack of proprioception influences gait control as represented e.g. by a more conservative gait pattern in diabetic PNP patients while demanding attention resources. Due to the reduced sensory information, gait control in diabetic PNP patients is more cognitively dependent than in healthy control individuals^30^. From a functional perspective, PNP patients typically walk more slowly, take shorter steps, and have greater gait variability than their age-matched peers^23,31–33^. In a previous study, we confirmed the PNP-related slowing of gait. Moreover, we found a relative speed reduction by about 13% in all major joints of the body during the walking sequence of the Timed Up and Go (TUG), including the arms^34^. Planning of movement components in the brain seems to run via velocity signals^35^. Those velocity signals are e.g. used in brain-machine interfaces to translate raw neuronal signals from the motor cortex into motor commands by means of velocity decoding^36,37^. Movements are represented as velocity vectors (vector metrics of direction and speed)^38,39^. We therefore assume that velocities are a suitable parameter for visualizing the limitations of PNP patients compared to age-matched healthy individuals.

The impact of PNP on goal-directed upper limb movements remains poorly understood. The function of the upper limbs is closely tied to many daily activities, and closely related to quality of life^40,41^ There are few studies, investigating the effect of PNP in upper limbs, showing lower accuracy and slower execution speed during goal-directed arm movements^42^as well as reduced functional hand performance during fine motor tasks compared to healthy individuals^43^.

It is assumed that cyclical movements and especially the coordination of limbs are regulated by a central mechanism (“central pattern generator”, CPG), and that this activity is regulated with the help of sensory feedback^3,44^. This mechanism appears to be particularly active during rhythmic movements (CPG-activity) where the upper limbs influence the reflex regulation of the lower limbs and their coordination^45,46^ and vice versa^1–3^. A previous study in healthy subjects revealed a significant correlation between the velocity of fast, repetitive, goal-directed arm movements (arm-movement test) and performance on the fast-executed TUG, independent of age^47^.

In this study, we compared the performances of PNP patients and healthy individuals during (i) fast repetitive goal-directed arm-movement (arm-movement test) and (ii) the fast executed TUG test. We assessed the duration to complete the TUG, gait speed, and whole body joint velocities during the walking sequences of the TUG. Arm-movement performance was evaluated by the number of cycles, frequency, and joint velocities of upper limbs, in a predefined time interval. We hypothesize that performance parameters of both test conditions correlate with each other, pointing to a more general impairment of movement execution in PNP.

## Materials and methods

### Participants

We recruited 20 patients diagnosed with clinically confirmed symptoms of PNP along with 20 healthy control participants (control group, CG) matched to patients‘ sex, height, age, and weight. Exclusion criteria were comorbidities that could affect gait and balance. Additionally, we asked about the number of falls during the last year and estimated the fear of falling via the validated Falls Efficacy Scale - International (FES-I)^48^. We also clinically assessed mobility performance by applying a common test, i.e., the Performance Oriented Mobility Assessment (POMA), with nine items for balance (score 0–16) and eight items for gait (score 0–12): a lower score indicates a higher risk of falling^49^ (see Table 1., see also^34^.

**Table 1:** Participants’ characteristics.

	PNP	matched CG	*p*-value
	*n* = 20	*n* = 20	
**Age** mean ± SD	60.7 ± 13.9	60.4 ± 14.7	.939^1^
**Sex** (m: f) N (%)	15:5 (75:25)	15:5 (75:25)	1.000^2^
**BMI** (kg/m²) mean ± SD	26.9 ± 5.2	25.2 ± 3.9	.264^1^
**Falls** [past year] N **Faller/non-faller** N (%)	32 13 (65)/7 (35)	2 2 (10)/18 (90)	**0.001** ^**3**^
**FES-I** [16–64 Points] mean (range)^a^ - Low concern [16–19] N (%) - Moderate concern [20–27] N (%) - High concern [28–64] N (%)	22.1 (16–38) 9 (45) 6 (30) 5 (25)	17.4 (16–20) 16 (80) 4 (20) 0 (0)	**0.006** ^**3**^
**POMA** [0–28 Points] mean (range)b - Moderate risk of falling [19–24] N (%) - High risk of falling [10–19] N (%)	22.6 (10–28) 6 (30) 5 (25)	27.8 (27–28) 0 (0) 0 (0)	**< 0.001** ^**3**^
**PNP entity** N (%) - CIPN - CIDP Not classified	4 (20) 15 (75) 1 (5)		
**Disease duration** (months) mean (range)	52.5 (8–256)		
**PNP specific treatment** N (%) - Rituximab - IVIg	2 (10) 14 (70)		
**PNP sensorimotor deficits N of 4 (%)** - No sensory deficits - 1 deficit - 2 deficits - 3 deficits - 4 deficits - Reduced reflexes AT/PT - Loss of reflexes AT/PT	4 (20) 7 (35) 2 (10) 5 (25) 2 (10) 10 (50)/6 (30) 4 (20)/8 (40)		

PNP, polyneuropathy patients; matched CG, matched healthy control group; BMI, Body Mass Index; FES-I, Falls efficacy Scale-International; POMA, Performance Oriented Mobility Assessment; SD, standard deviation; 1 T-Test; ² Pearson-Chi-Quadrat; ³ Man-Whitney-U, a Classification from Delabaere et al. (2010); b Classification from Tinetti, Speechley & Ginter (1988); significant differences (*p* < 0.05) between groups are marked in bold.

PNP, polyneuropathy patients; CIPN, chemotherapy induced polyneuropathy; CIDP, chronic inflammatory demyelinating polyneuropathy; IVIg, intravenous immunoglobulin infusions; Sensory deficits (contains measurement of vibration sense, position sense, temperature sensation, pain sensation); AT, Achilles tendon; PT, Patella tendon.

This study was approved by the Ethics Committee of the University of Freiburg (No. 68/19) and conducted according to the Declaration of Helsinki (German Register of Clinical Trials No.: DRKS00016999). Written informed consent was obtained from all individual participants included in the study.

### Assessments

Both the Timed Up and Go test (TUG)^50^ and the fast repetitive goal-directed arm-movement test (arm-movement test) were recorded using a markerless camera-based system, i.e., The Captury (The Captury GmbH, Saarbrücken, Germany). Data of whole-body movements was recorded with 12 cameras at 100 Hz. Participants were instructed to stand up from a standard-height chair, walk a distance of three meters (marked with tape on the floor), turn around, walk back, and sit down again, as quickly and safely as possible without running^63^. The test was performed twice while participants wore their own footwear. The arm-movement test was conducted while the participants were seated. They were instructed to alternately touch two platforms positioned 26 cm apart with each hand as quickly as possible for a duration of 20 s. Each limb was tested separately in two trials. The mean velocity across left and right limbs was calculated and used for further analysis. Data processing.

**Fig. 1:**
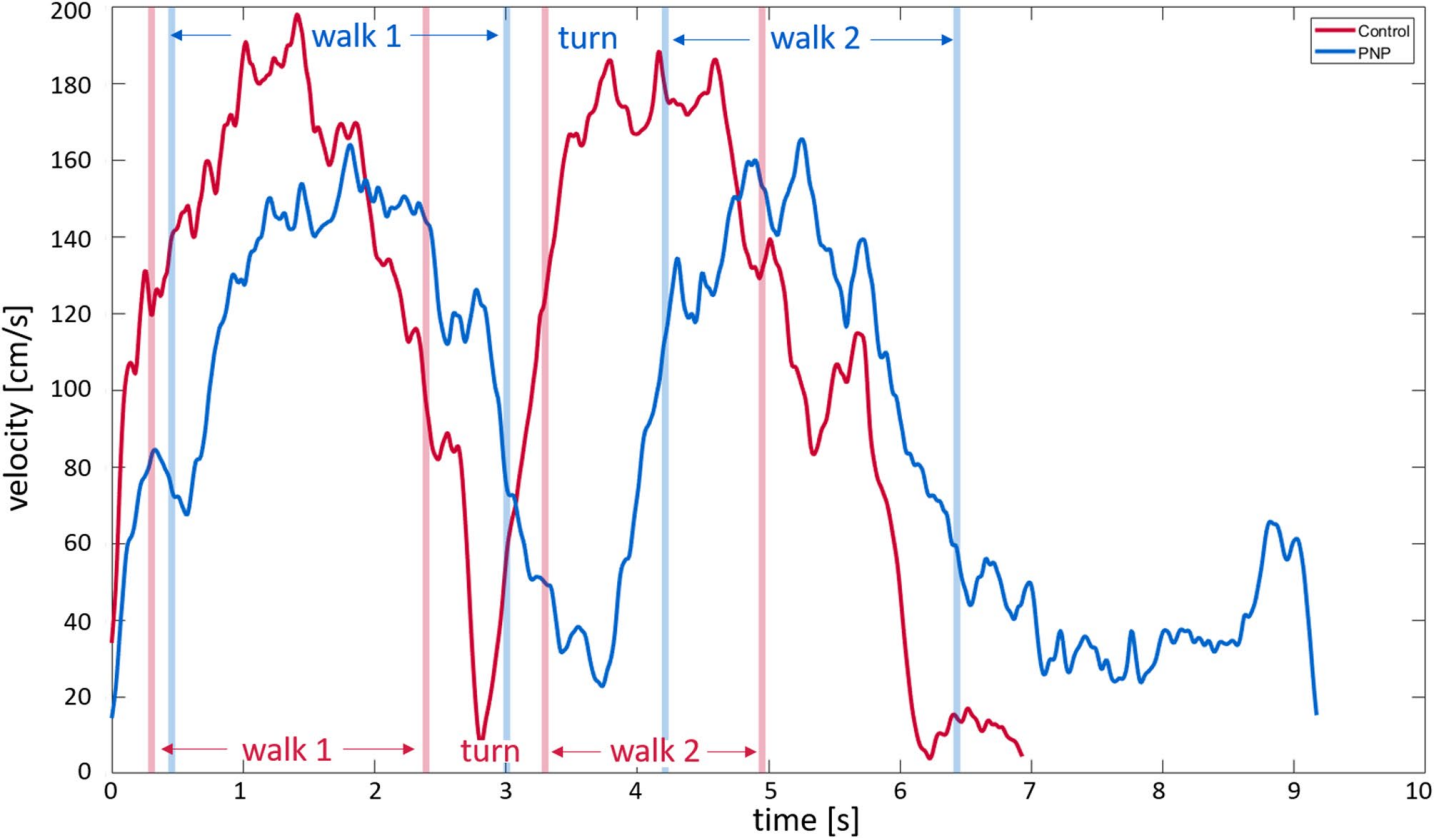
Velocity profile of the center of mass during the timed up and go test. The figure displays the COM velocity profile of one representative healthy control individual (red) and one PNP patient (blue). X-axis represents the time to complete the TUG test, y-axis the velocity [cm/s]. Vertical lines indicate a segmentation point e.g. walk 1, turn, walk 2.

A custom build MATLAB™ (R2019b; MathWorks, Natick, Ma) program was used for data processing. For analysis, mean values were used for the fast TUG and arm-movement test. To enable a detailed analysis of TUG performance, the TUG was segmented into three distinct phases: (1) Walk 1 – the walking segment between the stand-up and the turn, (2) Turning – the 180° turning phase, and (3) Walk 2 – the walking segment between the turn and the turn-to-sit task. For velocity-related measures, the mean velocity was calculated by averaging the values from Walk 1 and Walk 2. Turning and turn-to-sit sequences were identified based on shoulder axis rotation exceeding 20°^34^. Figure 1 illustrates the segmentation points for an example participant with PNP and an example healthy control, showing the velocity traces of the center of mass (COM) during the fast TUG. COM velocity refers to space coordinates and thus represents gait speed [cm/s]. Joint velocities (wrist, elbow, shoulder, hip, knee, and ankle) were calculated relative to COM during the walking sequences (Walk 1 and Walk 2). Velocity parameters [cm/s] were calculated as the mean between left and right joint velocities and the mean values of Walk 1 and Walk 2, as there was no statistical difference between them.

**Fig. 2:**
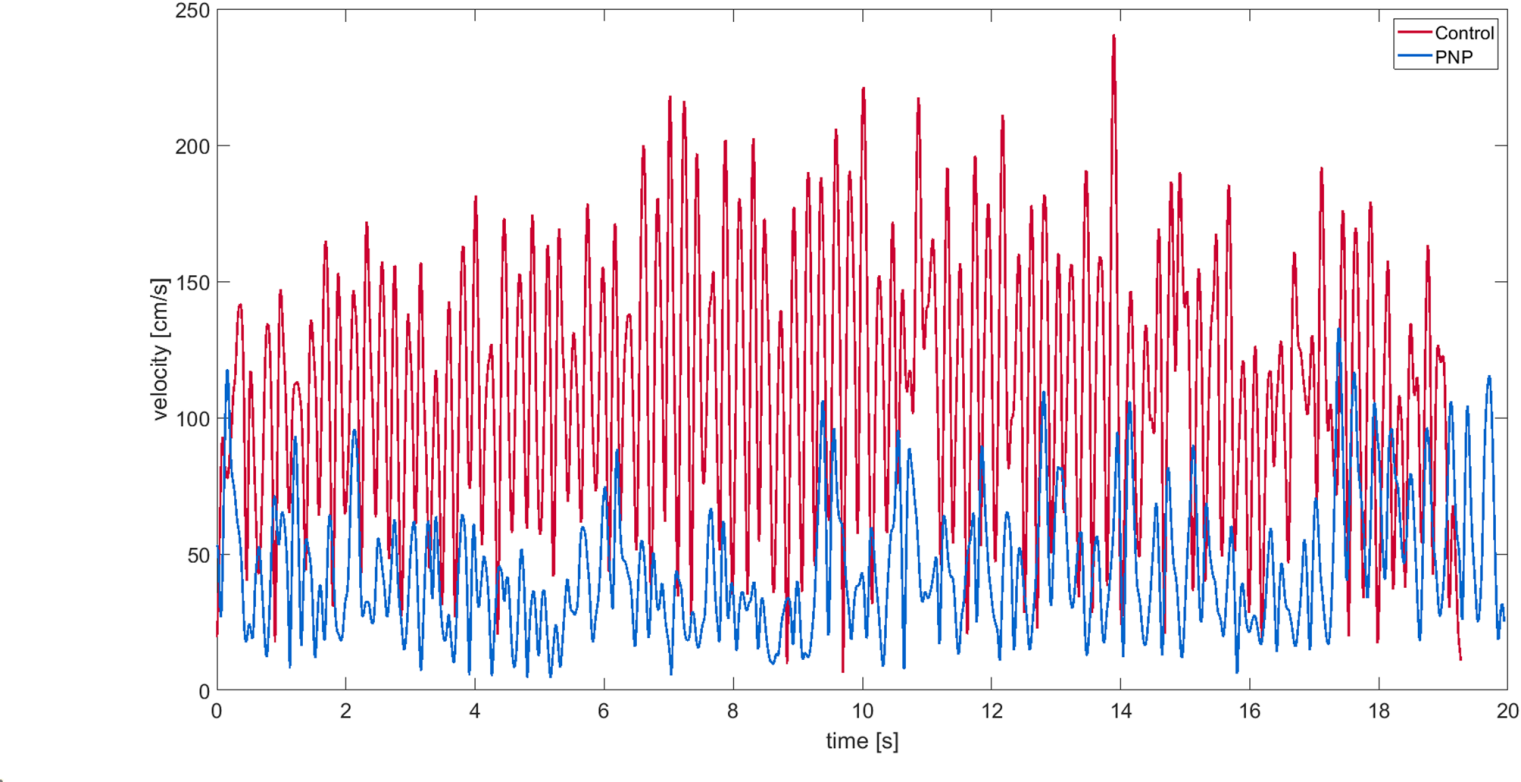
Velocity profile of the arm-movement test. The figure shows the velocity profile of one representative healthy control individual (red) and one PNP patient (blue). X-axis represents the 20 s period of the arm-movement test, y-axis the velocity of the wrist [cm/s].

For analysis of the arm-movement tests, we calculated mean and maximum joint velocities [cm/s] of wrist, elbow and shoulder in room coordinates. We identified the start and end of a cycle by the maximum distance predetermined by the distance of the target platforms (26 cm). One cycle consists of one touch left and right to left again (Fig. 2 displays velocity traces of the wrist for an example participant with PNP and an example healthy control). We extracted frequency [Hz], amounts of cycles [N], and fatigue [% change from the first 12 to the last 12 cycles].

#### Statistical analysis

For statistical analysis, IBM SPSS Statistics for Windows, version 26.0 (IBM Corp., Armonk, N.Y., USA), and for data visualization, RStudio, version 4.3.1 (RStudio, PBC, Boston, USA) was used. Descriptive statistics are reported as median with the interquartile range (IQR 25–75 percentile). The Shapiro-Wilk test was used for normal distribution of arm-movement parameters (all parameters were normal distributed, except cycles and frequency in PNP). As there were no significant differences between left and right limbs, we used the mean of both sides for analysis (*N* = 40; CG = 20, PNP = 20 for each joint, cycles, frequency).

Differences between groups during the arm-movement test (parameters: cycles and frequency) and walking sequence of the fast TUG test (parameters: gait speed and TUG time) were analyzed separately using repeated measures ANOVA^51^. For the analysis of the arm-movement test parameters (joint velocity, maximum joint velocity and fatigue), joint (wrist, elbow, shoulder) and for fast TUG test parameter (joint velocity), joint (wrist, elbow, shoulder, hip, knee, ankle) was added as second variable (two-way repeated measures ANOVA). Bonferroni was used as a post-hoc test for significant results. Comparisons of velocity curves were conducted using SPM12, an open-source software package for statistical parametric mapping (https://www.fil.ion.ucl.ac.uk/spm/software/spm12/). Joint velocity traces were compared individually using the paired t-test function of the SPM12 software. To illustrate group differences, a regression plot was generated comparing the PNP group and healthy controls, based on mean joint velocities from the arm-movement test and fast TUG test. To quantify the slowing in PNP patients relative to healthy controls, velocity ratios were calculated by dividing velocities of the PNP group by those of the control group.

Correlations between parameters of the fast TUG test and the arm movement test were analyzed using segmental and intersegmental regression models based on joint velocities. Analyses were conducted separately for the PNP group and the control group. Segmental associations referred to the relationship between upper limb joint velocities (wrist, elbow, shoulder) obtained during the fast TUG test and those from the arm-movement test. Intersegmental associations examined the relationship between lower limb joint velocities (ankle, knee, hip) during the TUG test and upper limb joint velocities (wrist, elbow, shoulder) during the arm-movement test. Velocity ratios between groups for the (TUG test and the arm-movement test (AMT)) were then correlated with the TUG time [s], gait speed [cm/s], fear of falling (FES-I) and mobility performance (POMA) using the Spearman-Rho test (ρ).

## Results

No adverse events occurred during the tests and all participants performed all test conditions. We included data from *N* = 40 participants (20 PNP: 20 CG) in our analysis. As shown in Table 1, matching was performed adequately with no significant differences between participants’ age, sex, height and weight (displayed as BMI). There were significantly more falls and faller in the PNP group, with higher fear of falling (FES-I) and lower scores in the POMA in the PNP group. The most common PNP entity was a chronic inflammatory demyelinating polyneuropathy (CIDP, 75%), followed by chemotherapy induced polyneuropathy (CIPN, 20%) and only one case of an idiopathic origin. 80% of PNP patients received PNP-specific treatment.

Arm-movement test.

Analysis of joint velocities (two-way repeated ANOVA, Table 2) during the arm-movement test revealed a significant influence of the factors ‘group designation’ (PNP, CG; F = 19.47, *p* = 0.030, η^2^ = 0.224, Fig. 4A) and ‘joint’ (wrist, elbow, shoulder, F = 473.93.52, *p* < 0.001, η^2^ = 0.981, Fig. 4B-D). Post-hoc test for joint velocities revealed a significant difference between all 3 joints (wrist vs. elbow: difference 52.1 cm/s, *p* < 0.001; elbow vs. shoulder: difference 57.0 cm/s, *p* < 0.001; wrist vs. shoulder: difference 109.0 cm/s, *p* < 0.001).

**Table 2:** Repeated measures ANOVA and two-way repeated measures ANOVA results for arm-movement and fast TUG test parameters.

Parameter	Group (PNP, matched CG)	Joint ^1^	Group * joint
Arm-movement test			
Joint velocity [cm/s]	F = 19.47, *p* = 0.030	F = 473.93, *p* < 0.001	F = 2.10, *p* = 0.151
Max. Joint velocity [cm/s]	F = 4.46, *p* = 0.048	F = 799.06, *p* < 0.001	F = 1.30, *p* = 0.296
Fatigue [%]	F = 0.33, *p* = 0.569	F = 1.73, *p* = 0.206	-
Cycles [N]	F = 10.69, *p* = 0.002	-	-
Frequency [Hz]	F = 16.16, *p* < 0.001	-	-
**Fast TUG test**			
Joint velocity [cm/s]	F = 7.82, *p* = 0.011	F = 135.31, *p* < 0.001	F = 2.47, *p* = 0.080
Gait speed [cm/s]	F = 13.05, *p* = 0.002	-	-
TUG time [s]	F = 17.36, *p* < 0.001	-	-

PNP, polyneuropathy patients; matched CG, matched healthy control group^1^, joints arm-movement test -wrist, elbow, shoulder; joints TUG test - wrist, elbow, shoulder, hip, knee, ankle.

**Fig. 3:**
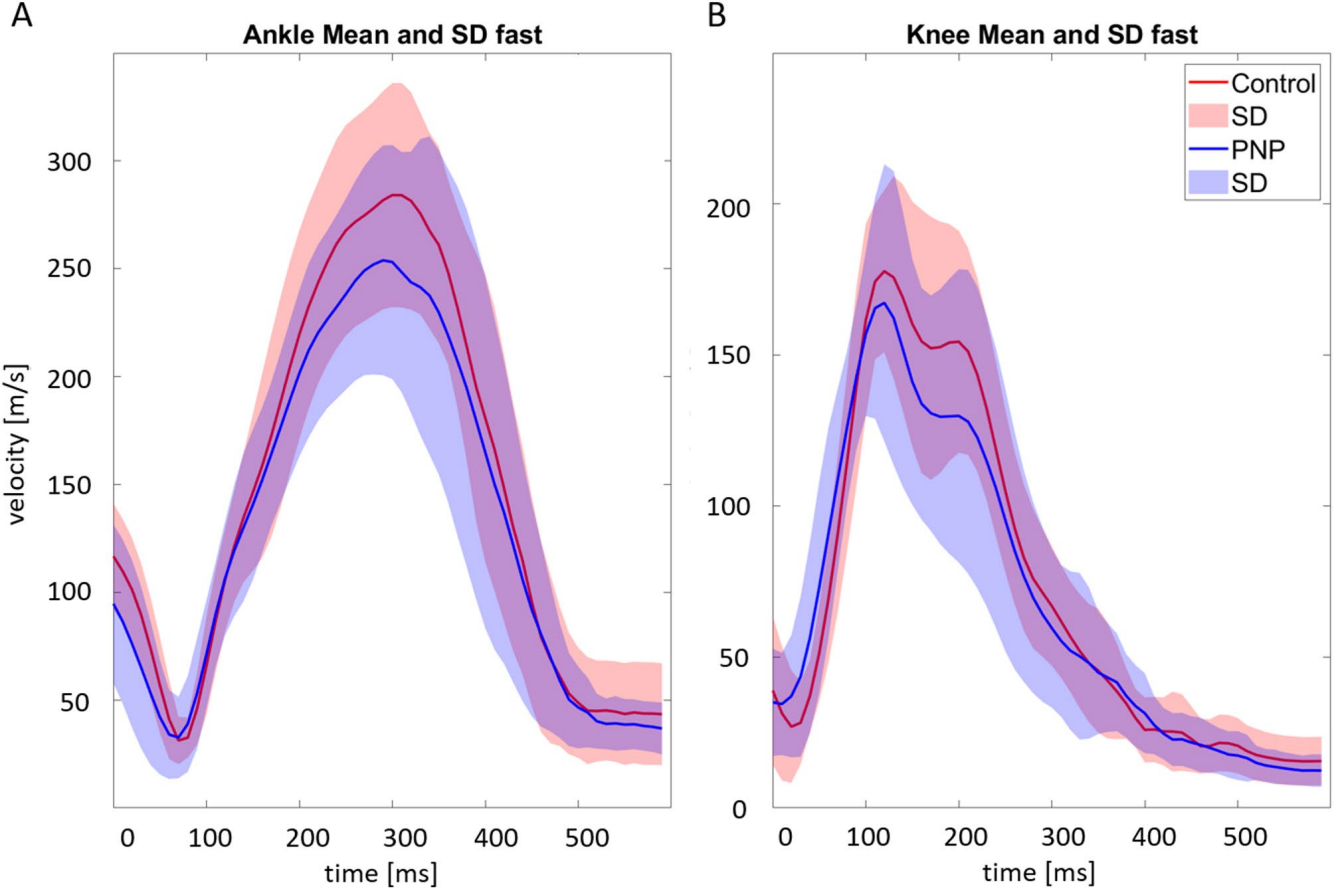
Joint speed traces ankle and knee. The figure presents the mean joint velocity profiles for a step (solid lines) and corresponding standard deviations (shaded areas) for the ankle (**A**) and knee (**B**) during the fast TUG test. Data are plotted over time (x-axis, in milliseconds), with joint velocity shown on the y-axis (in m/s). Two groups are compared: healthy controls (red) and individuals with Polyneuropathy (PNP) (blue).

Maximal joint velocities revealed a significant influence of the factors ‘group designation’ (PNP, CG; F = 4.46, *p* = 0.048, η^2^ = 0.190) and ‘joint’ (wrist, elbow, shoulder, F = 799.06, *p* < 0.001, η^2^ = 0.989). Post-hoc test for maximum joint velocities revealed a significant difference between all three joints (wrist vs. elbow: difference 84.0 cm/s, *p* < 0.001; elbow vs. shoulder: difference 135.2 cm/s, *p* < 0.001; wrist vs. shoulder: difference 219.1 cm/s, *p* < 0.001). Maximal joint velocity was largest in the wrist and slowest in the shoulder, as expected.

Fatigue was not statistically different neither for group designation (PNP, CG; F = 0.33, *p* = 0.569, η^2^ = 0.017) nor for joint (wrist, elbow, shoulder, F = 1.73, *p* = 0.206, η^2^ = 0.161). PNP patients performed significant less cycles within the 20 s (F = 10.69, *p* = 0.002) with a lower frequency (F = 16.16, *p* < 0.001).

### Fast TUG test

Analysis of joint velocities (two-way repeated ANOVA, Table 2) during the walking sequence of the fast TUG test showed a significant influence of the factors ‘group designation’ (PNP, CG; F = 7.82, *p* = 0.011, η^2^ = 0.292) and ‘joint’ (wrist, elbow, shoulder, hip, knee, ankle F = 135.31, *p* < 0.001, η^2^ = 0.978). There was no interaction between the factors group and joint (*p* = 0.08).

When comparing mean joint speed traces of relevant joints between PNP patients and CG (ankle, knee), we found an almost evenly distributed reduction of joint speed along joint movement pieces associated with single steps, leading to larger step durations (Fig. 3).

**Fig. 4:**
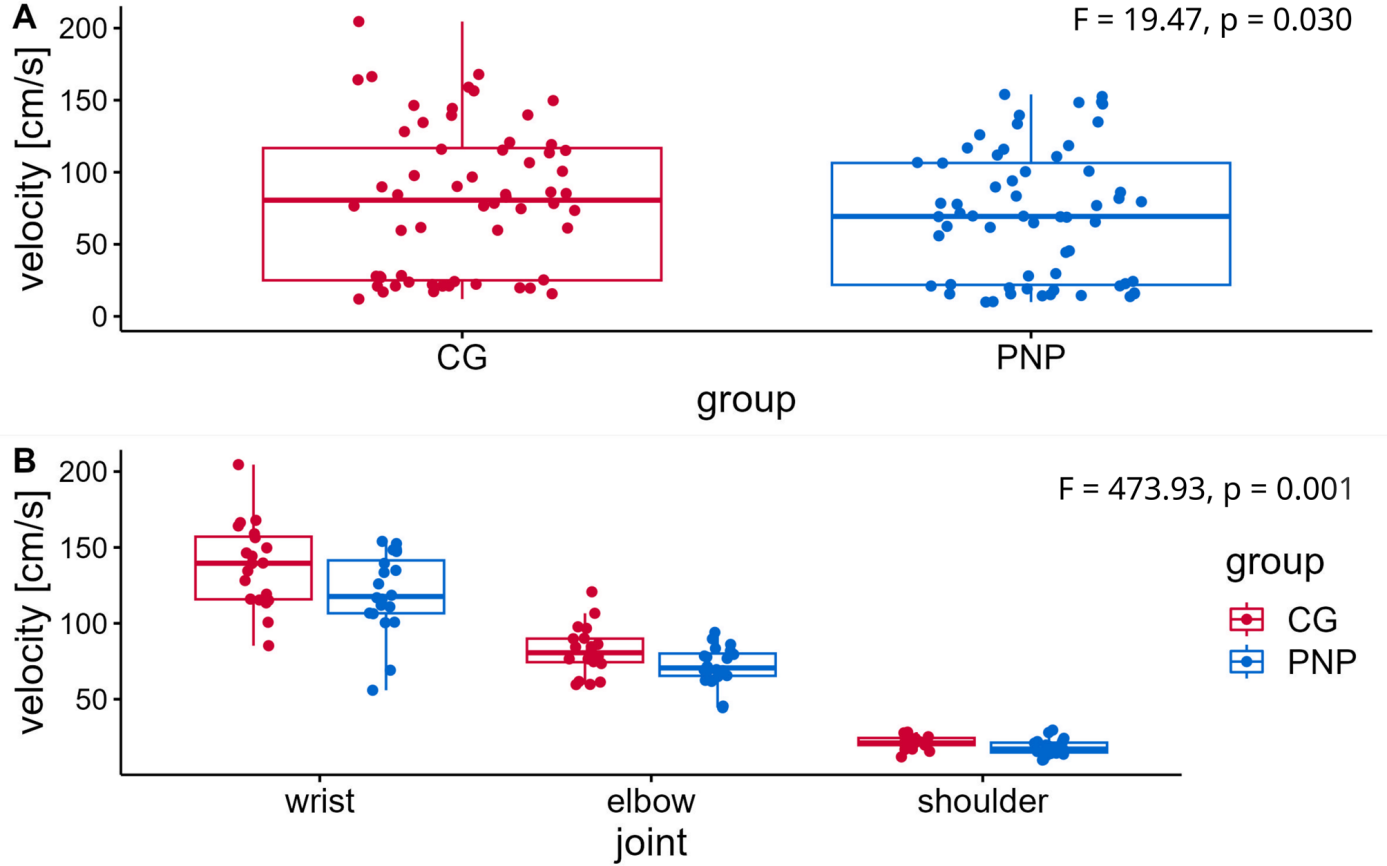
Mean joint velocities of upper limbs. The figure shows the mean joint velocities of the upper limbs (in space coordinates) (y-axis) from the arm-movement test per group (CG (red), control group (*n* = 60: wrist = 20, elbow = 20, shoulder = 20); PNP (blue), polyneuropathy patients (*n* = 60: wrist = 20, elbow = 20, shoulder = 20)(A) mean joint velocity across all joints (wrist, elbow, shoulder) for CG and PNP group; (B) mean joint velocity per joint for CG and PNP group. Boxplots showing the lower quartile (25th percentile), median (50th percentile), upper quartile (75th percentile), and degree of dispersion as 95% confidence interval (95% CI) (whiskers), outliers are marked with an x. F-statistic for two-way repeated measures ANOVA is shown in the upper right corner for (A) group differences and (B) joint velocities.

Gait speed (F = 13.05, *p* = 0.002), and TUG time (F = 17.36, *p* < 0.001) also significantly differ between PNP patients and CG (Table 2).

### Correlation analysis

**Fig. 5:**
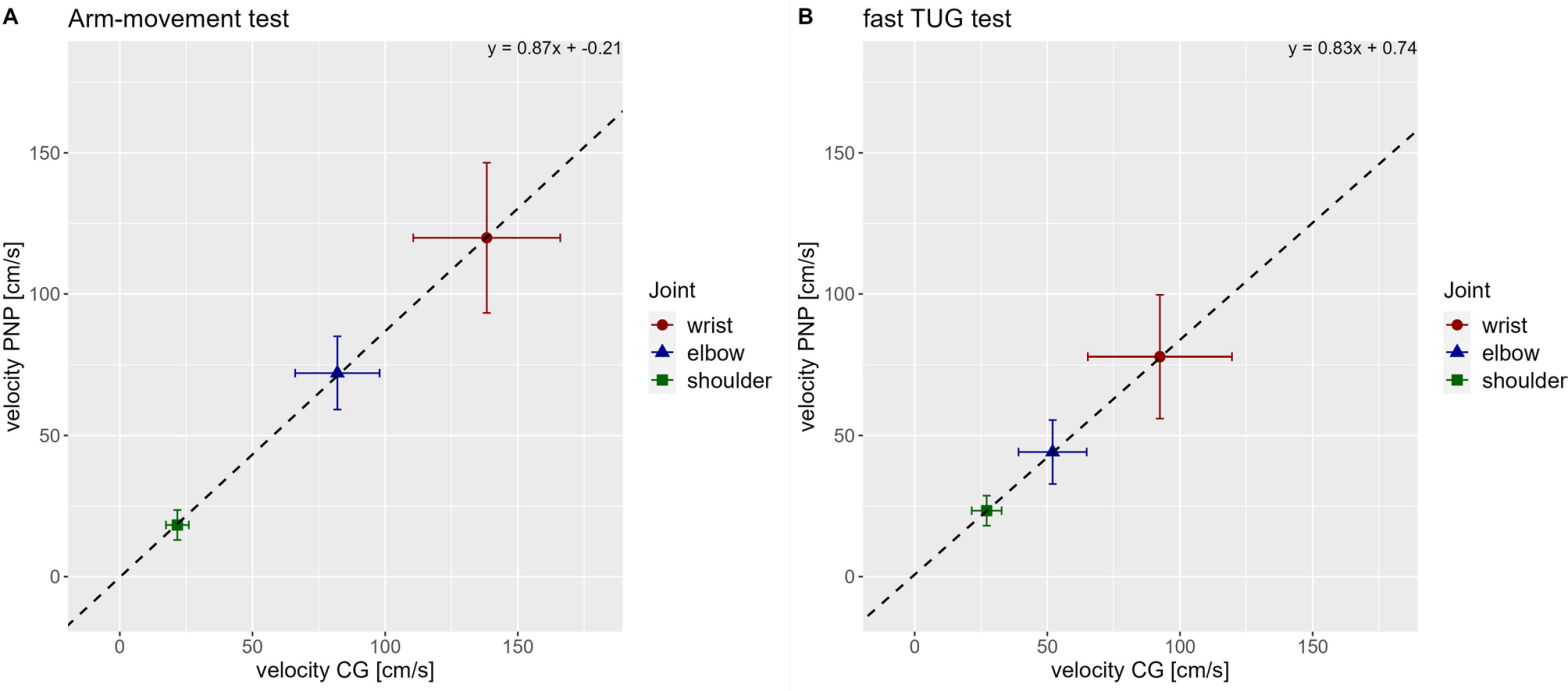
Velocity ratios between groups. (**A**) Correlation between mean joint velocities (wrist (red), elbow (blue), shoulder (green)) of PNP group (y-axis) and CG (x-axis) for the arm-movement test, including the regression equation. (**B**) Correlation between mean joint velocities (wrist (red), elbow (blue), shoulder (green)) of PNP group (y-axis) and CG (x-axis) for the walking sequence of the fast executed TUG test, including the regression equation. Boxes and whiskers show the mean value of the velocity (box) and standard deviation (horizontal whiskers for CG, vertical whiskers for PNP group).

We plotted mean velocities for wrist elbow and shoulder during the arm-movement test and the TUG test of PNP against CG (for descriptive values see Table 3). Figure 5A displays the three joint velocities during the arm movement test, Fig. 5B during the TUG test. Both tests revealed a ratio between groups with 0.87 (Fig. 5). Joint velocities of PNP were reduced overall by 13% compared to CG independently of joint and test.

**Table 3:** Descriptive values of PNP and matched CG for repetitive goal-directed arm-movement test and fast TUG.

	Joint	PNP median (IQR)	matched CG median (IQR)
**Arm-movement test**		*n* = 20	*n* = 20
**Velocity [cm/s]**	Wrist	117.7 (106.4–145.4)	139.6 (115.5–158.4)
	Elbow	70.6 (65.1–81.3)	80.6 (73.8–90.1)
	Shoulder	17.2 (14.6–21.8)	21.2 (19.6–25.0)
**Max. Velocity [cm/s]**	Wrist	255.4 (203.8–294.4)	280.8 (250.2–308.5)
	Elbow	169.7 (150.8–195.0)	190.9 (175.3–212.1)
	Shoulder	43.4 (35.4–51.1)	50.0 (46.0–60.4)
**Fatigue [%]**	Wrist	4.9 (0.5–8.2)	4.9 (2.2–7.5)
	Elbow	4.9 (0.7–9.0)	2.8 (−0.1–6.3)
	Shoulder	1.1 (−0.6–6.2)	5.3 (1.5–9.1)
**Cycles [N]**		37.4 (21.2–45.0)	42.3 (38.5–47.5)
**Frequency [Hz]**		0.52 (0.45–0.58)	0.47 (0.41–0.51)
**Fast TUG**		*n* = 20	*n* = 20
**Velocity [cm/s]**	Wrist	79.8 (60.4–93.0)	88.3 (70.1–112.9)
	Elbow	43.2 (33.7–50.9)	49.8 (40.5–63.9)
	Shoulder	22.3 (19.0–27.2)	25.7 (23.3–30.0)
	Hip	20.9 (17.6–24.6)	22.9 (19.9–25.9)
	Knee	78.9 (62.7–97.8)	82.0 (76.7–104.7)
	Ankle	136.6 (96.5–154.7)	148.6 (133.9–163.0)
**Gait speed [cm/s]**		134.7 (96.5–166.2)	148.8 (134.1–175.0)
**TUG time [s]**		8.2 (6.2–10.9)	6.5 (5.8–7.0)
**Walk 1 [s]**		2.4 (1.9–3.4)	1.9 (1.6–2.2)
**Turning [s]**		1.1 (0.9–1.7)	0.9 (0.9–1.0)
**Walk 2 [s]**		2.3 (1.8–3.1)	1.9 (1.6–2.0)

PNP, polyneuropathy patients; matched CG, matched healthy control group; IQR, interquartile range (25–75 percentile).

**Fig. 6:**
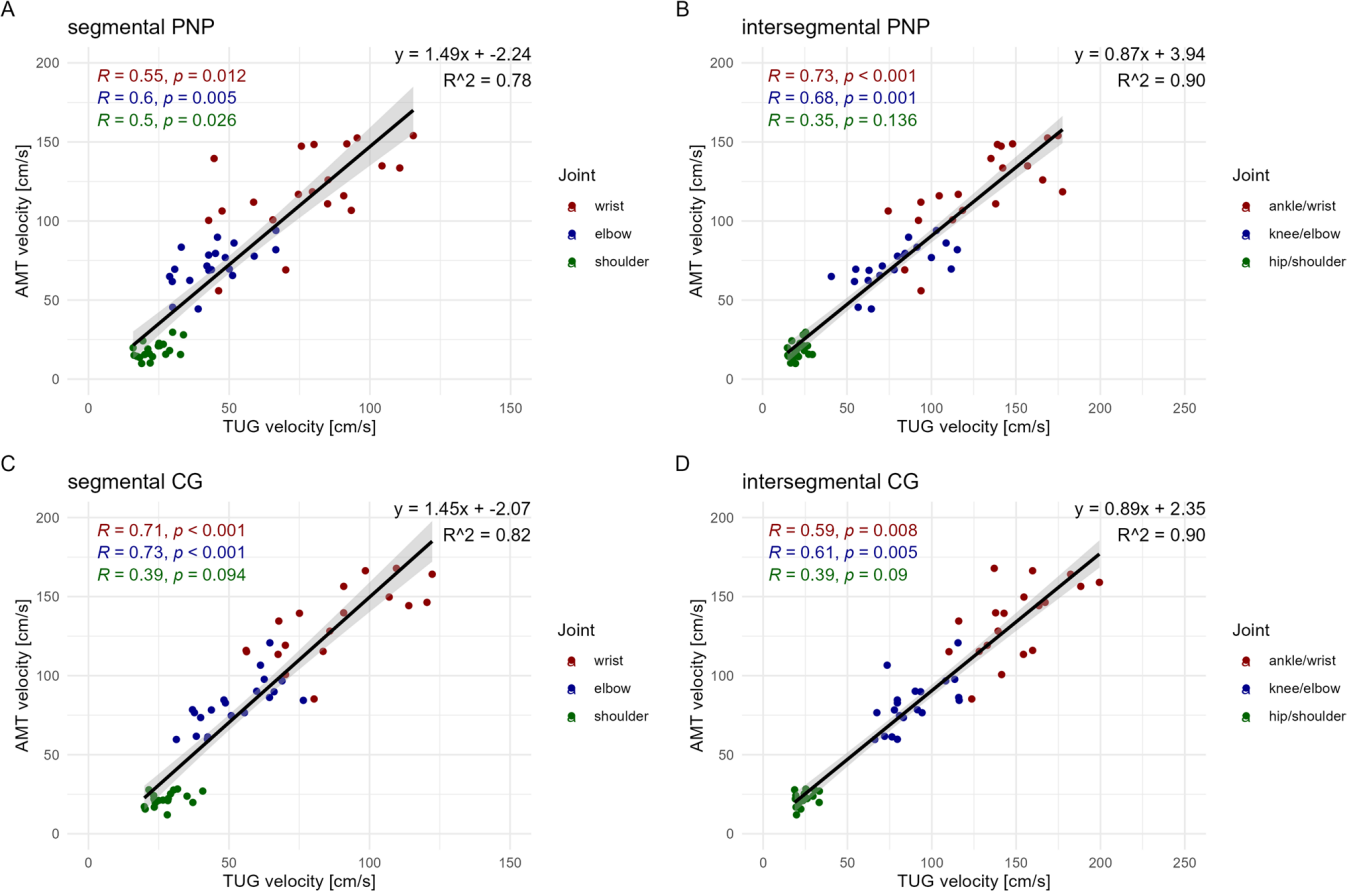
Segmental and intersegmental correlations. The figure illustrates the correlation between the joint velocities (segmental: wrist, elbow, shoulder; intersegmental: ankle, knee, hip) during the fast TUG test (x-axis) and the joint velocities (wrist, elbow, shoulder) of the arm-movement test (AMT) (y-axis). A distinction is made between segmental (**A**, **C**) and intersegmental (**B**, **D**) analyses, as well as between patients with Polyneuropathy (PNP; **A**, **B**) and healthy controls (CG; **C**, **D**). The represented joints (wrist, elbow, shoulder or their intersegmental combinations wrist/ankle, elbow/knee, shoulder/hip) are color-coded. Correlation coefficients (R) and corresponding p-values are reported separately for each joint.

As displayed in Fig. 6, across all panels, a positive correlation between TUG velocity and arm-movement test joint velocities is observed. The strongest correlations were found in the intersegmental analysis within the PNP group (Fig. 6B; R² = 0.90) and in the segmental analysis within the control group (Fig. 6C; R² = 0.87). Notably, shoulder movements (green) exhibited weaker or non-significant correlations compared to more distal joints (wrist and elbow).

**Fig. 7:**
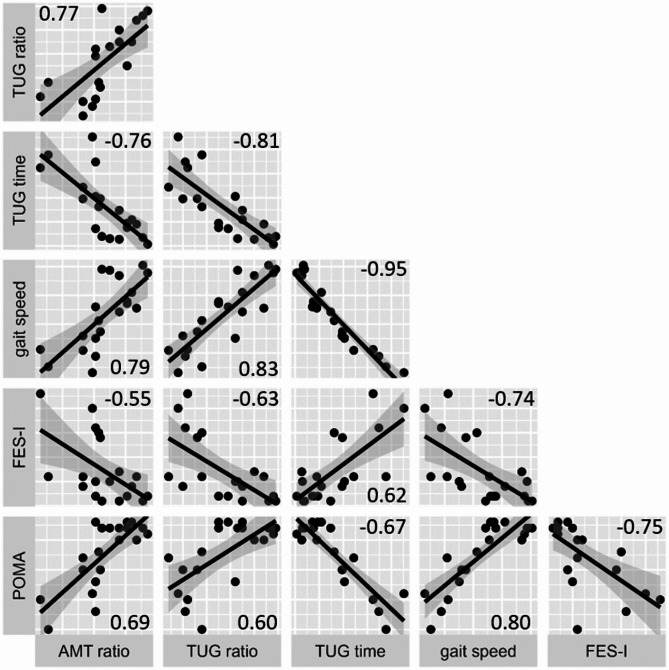
Correlations between velocity ratios, tug performance, and clinical scales. Results of the spearman correlation between the velocity ratios (CG/PNP mean velocities) of the fast TUG test (TUG ratio) and the arm-movement test (AMT ratio) with the time to complete the fast TUG, gait speed during the walking sequence of the fast TUG and clinical scales (Falls efficacy Scale-International, FES-I; Performance Oriented Mobility Assessment, POMA). Lower triangular part of the correlation matrix displays correlation plots of the PNP data, with the extent of the correlation (Spearman-Rho, ρ).

The correlation analysis showed that the overall velocity ratio of the TUG and the overall velocity ratio of the arm movement test are highly positive correlated (ρ = 0.773, *p* < 0.001). Velocity ratio of the arm-movement test correlates negatively with TUG time (ρ = −0.758, *p* < 0.001) and fear of falling (ρ = −0.552, *p* = 0.012), positively with gait speed (ρ = 0.788, *p* < 0.001) and mobility performance (ρ = 0.688, *p* = 0.001). A higher fear of falling and poorer mobility were associated with slower velocities in the PNP group (Fig. 7).

## Discussion

The present data analysis has reproduced the relative slowing of joint movements in PNP patients during gait intervals of the TUG, while being instructed to act “fast”. More specifically, PNP patients show a relative reduction in movement speed about 13% across all major joints of the body, irrespective of the individual joint speed level. In other words, the relatively low hip speed is reduced by a similar percentage as the relatively high foot or hand speed. Moreover this speed reduction in single joints is nearly evenly distributed across joint trajectories, leading, in turn, to longer-lasting joint movements, when focusing on data pieces segmented for single step analysis.

In addition, we demonstrate that PNP patients show a slowing of those joints that are involved in arm movements in a totally different task, i.e. repetitive goal-directed arm movements between two goals, while sitting. The speed reduction appeared in both, mean and maximum, joint velocities. In parallel to the arm speed reduction, PNP patients showed fewer cycles of arm movements in a given time. Correlation analyses between mean joint velocities during the goal-directed arm movements and during the walking sequences of the fast TUG revealed a strong correlation between joint velocities of the two movement tasks. Considering velocity ratios between groups, both tasks revealed a reduction of upper limb joints’ velocities (wrist, elbow, shoulder) in PNP compared to CG with a ratio of 0.87. In regard of clinical tests, the reduced joint velocities in PNP strongly correlated with the time to complete the TUG test, gait speed, FES-I and POMA. These findings underscore that generalized slowing may be a fundamental feature of motor impairment in PNP patients.

Slowing seems to be the most characteristic feature of PNP patients’ movement behavior. Studies assessing gait speed in PNP patients typically report a slowing of gait speed of 20–30% compared to healthy individuals^15,52,53^. This slowing is also reflected in the duration of the TUG test, which took PNP patients around 13.4 s^54–58^with a cut-off value of < 13.5 s indicating a high fall risk^59,60^. PNP patients also show a relative slowing of gait independently of the instruction (walk at preferred, fast speed^33^or while executing an additional (e.g. cognitive) task^34^.

While there is a substantial body of literature documenting overall gait slowing in various polyneuropathy entities^32,33,61,62^there is limited research on the distribution of velocities across different body joints including upper limbs^43,63^. Most studies that focus on upper limb function in PNP evaluated muscle strength, or reaching and grasping movements^41,63–67^. During gait, movements of the upper limbs contribute to the stabilization of the body and the efficiency of gait^68,69^ (e.g. generate propulsion while maintaining posture), a goal-directed arm movement necessitates a synergistic interaction between shoulder, elbow and wrist for a successful execution.

While there is some evidence for correlations between upper limb performance during isolated arm movement tasks, and walking e.g. in Parkinson’s Disease (PD) and in Multiple Sclerosis (MS)^70,71^this has not been demonstrated for PNP.

Based on the assumption that our findings are related to sensorimotor feedback control that typically integrates sensory inputs with motor outputs to achieve precise and adaptive actions, we speculate that PNP patients, like CG, are still able to modulate feedback gains by continuously adjusting the influence of sensory feedback on motor control. This modulation, known as sensory gain control, serves, in principle, various functions^72^: *(i)* enhancement of relevant feedback, i.e. amplifying feedback from pertinent sensory modalities to facilitate online corrections during movement, *(ii)* attenuation of disruptive feedback, i.e. diminishing the impact of irrelevant or self-generated sensory signals to maintain movement stability, and (iii) adaptation to environmental changes, i.e. adjusting feedback gains to accommodate novel task demands or altered environmental conditions. These adjustments are not static; they are flexible and context-dependent, reflecting the system’s capacity to optimize motor performance in real-time. We deem it likely that PNP patients attenuate their impaired proprioceptive sensory feedback, which may lead, in turn, to speed reduction of the motor output.

Moreover, sensorimotor control operates through a hierarchical framework, where multiple feedback loops function concurrently^73^. This includes low-level feedback representing immediate responses mediated by spinal circuits and muscle properties, intermediate-level feedback representing the processing in the cerebellum and brainstem that refines motor commands, and high-level feedback representing integration in cortical areas, such as the posterior parietal cortex (PPC), which combines sensory information with motor plans to guide goal-directed actions. This hierarchical structure allows for both rapid corrections and adaptable planning, ensuring that movements are both precise and flexible. We speculate that PNP patients’ velocity reduction comprises all three levels, since the impairment of proprioceptive feedback acts on all different loops.

From another perspective, internal models and predictive coding^74^ may play a role for the joint speed reduction in PNP: Internal models, particularly those involving the cerebellum and PPC, play a crucial role in predicting the sensory consequences of movements. While forward models, presumptively located in the cerebellum, predict the future state of the limb based on current motor commands, state estimation located in the PPC integrates sensory feedback with these predictions to assess the current state of the body and environment. This predictive framework enables the system to anticipate and correct errors promptly, enhancing the efficiency and accuracy of goal-directed movements. We deem it likely that this mechanism in itself is properly working in PNP patients. However, it might be adapted towards lower speed due to incorrect proprioceptive sensory inputs in order to avoid larger movement inaccuracies.

The significant correlation between goal directed arm movement joint velocities and the POMA as well as the FES-I score in PNP patients underscores the significance of the overall speed reduction as a facet of the altered sensorimotor control.

Understanding the altered sensorimotor feedback control in PNP patients may be vital for developing effective rehabilitation strategies, including motor learning, which may enhance the ability of PNP patients to re-weigh sensory inputs (see e.g^75^ *).*

## Data Availability

This article includes summarized data from this study. Raw data is available upon request (isabelle.walz@uniklinik-freiburg.de).
